# The experience of young adult cancer survivors’ engagement with nature

**DOI:** 10.1007/s11764-026-02008-2

**Published:** 2026-03-24

**Authors:** Erica R. Timko Olson, Donna Z. Bliss, Megan Driscoll, Angelica Walton, Ryne Wilson

**Affiliations:** University of Minnesota—Twin Cities, 308 SE Harvard St, Minneapolis, MN 55455 USA

**Keywords:** Cancer 1, Survivorship 2, Nature 3, Nature-based interventions 4, Wellbeing 5

## Abstract

**Background/objectives:**

Young adult (YA) cancer survivors, aged 18 to 39, face unique physical, emotional, and social challenges during critical developmental milestones. While nature-based engagement has potential as an intervention for these challenges, a research gap exists regarding the lived experiences of this population in nature-based engagement. The aim of this study was to describe YA cancer survivors’ experiences engaging with nature before and after a cancer diagnosis.

**Methods:**

A qualitative study was conducted with 15 YA cancer survivors recruited via social media and survivor groups. Data were collected through individual, one-on-one semi-structured interviews via Zoom, audio-recorded and transcribed. Data were analyzed iteratively using a general inductive approach and managed in NVivo to identify emergent themes.

**Results:**

Participants were predominantly female, white, and diagnosed with breast cancer (mean age at diagnosis = 30.5 years). Identity, Reconstruction, and Healing emerged as the overarching central theme. Four main themes were as follows: (1) nature as a sanctuary for therapeutic and spiritual reflection, (2) transformation of the survivor’s relationship with nature, (3) the role of nature in reclaiming agency, and (4) barriers to being in nature during illness.

**Conclusions:**

Findings support nature’s vital role as a restorative resource for emotional and physical recovery for YA cancer survivors. Results support further research into modalities of nature-based interventions that promote their social interaction and reduce barriers to engagement, including virtual reality-based nature interventions.

**Implications for Cancer Survivors:**

Nature offers opportunity for YA cancer survivors to reconcile their pre- and post-diagnosis identities and help improve their well-being and quality of life.

## Introduction

Young adult (YA) cancer survivors, typically defined as individuals aged 18 to 39, face distinct challenges as they navigate life during and after treatment. Jacobs [1] found 91% of pediatric, adolescent, and young adult cancer survivors reported at least one late-term effect of treatment that included fatigue (73%), concentration (57%), body image problems (48%), anxiety (21%), and depression (9%), which attributed to worse psychosocial outcomes. In response to these challenges, nature-based engagement has emerged as a promising intervention to improve the psychological and physical well-being of young adults [2, 3]. YA cancer survivors experience a range of physical, emotional, and social difficulties that are unique to their stage of life [4] and often struggle with long-term health issues, including infertility, chronic pain, and psychological distress [5] as well as complexities of transitioning back to a sense of normalcy [6]. YA cancer survivors have lower quality of life scores, report a lack of control, have a different outlook on life than their peers, lack peer support, and feel different from their peers, which is a source of stress, uncertainty, and worry [7, 8].

Nature-based practices have been associated with positive health effects in healthy adults, including a reduction in cortisol levels by 15.8% on average, a 3.9% lower heart rate, a 2.1% decrease in blood pressure, and enhanced parasympathetic nerve activity of 102% after walking in a forest setting [9, 10]. Nature-based therapies support immune system function, decrease depression and anxiety, and promote mental relaxation, gratitude, and selflessness [11]. However, there is less information about the effects of nature on the cancer survivor population [12]. A survey of 2355 adults and older adults with cancer reported nature was the most important coping strategy and a resource in dealing with their disease during their cancer experience. Interactions with nature made them feel better when they were sad, stressed, or depressed and gave them a spiritual sense [13]. In a review of 16 peer-reviewed research articles, Ray and Jakubec [14] found that nature interventions with cancer survivors are associated with decreased fatigue and anxiety and increased sense of belonging and self-esteem. Forest therapy has been shown to improve sleep quality among patients with gastrointestinal tract cancers [15].

As many of these findings are from studies of adults, there is a notable gap in research specifically addressing the experiences of YA cancer survivors with nature-based interventions. One study found that YA cancer survivors identified nature-based therapies as promoting relaxation and self-reflection [16]. Another noted nature-based intervention has been linked to improved social outcomes, fostering feelings of connection and support among cancer survivors [17]. As the understanding of cancer survivorship evolves, shifting from a focus on merely “surviving cancer” to overall well-being [18], it is crucial to explore the role of nature-based engagement in the well-being of YA cancer survivors. The aim of this study is to describe YA cancer survivors’ experiences engaging with nature before and after their cancer diagnosis.

## Materials and methods

### Design, recruitment, and eligibility

This study has a qualitative design. Participants, aged 18–39 years, were purposively recruited from local cancer survivor groups via email listservs and social media: Taking Charge of your Survivorship, through the Earl E. Bakken Center for Spirituality and Healing, the Masonic Cancer Center, and Gilda’s Clubhouse Minnesota. Prospective participants were provided with a link to a screening survey using REDCap software [19] to ensure that inclusion criteria were met. Inclusion criteria were being between the ages of 18–39 years at time of cancer diagnosis, and at time of study consent, and less than 5 years since cancer treatment ended. The PI obtained informed consent from the participants online via Zoom. The PI reviewed the consent with the participant, ensuring comprehension by having the participant explain the purpose and their role in the study, before they signed electronically with the secure REDCap system, which included an agreement to an audio and video recorded interview. All people introduced to the study elected to participate. The Institutional Review Board of the University of Minnesota approved the study.

### Data collection

Demographic data were collected online, March–July 2022, through REDCap, following consent. The PI, a female PhD-prepared nurse, with advanced training in qualitative interviewing, conducted one-hour, individual semi-structured interviews with the participants via Zoom video conference. There was no relationship between the researcher and participants prior to the study. The participants were introduced to the researcher’s background and interests in the topic. See Table 1 for the Interview Guide that asked about the participants’ relationship to nature prior to, during, and following cancer treatment. Interviews were video and audio recorded, and the transcripts of each interview were autogenerated by Zoom and reviewed by two researchers for clarity. The clarified transcripts were then sent for approval to the participant. Recruitment concluded when saturation was reached; no new information was being shared by the participants.

**Table 1:** Interview guide

Question category	Questions asked
Cancer History	Diagnosis, Treatment and Age at diagnosis
Nature	When I use the word nature, what does that mean to you? What was your experience with spending time in nature before your cancer diagnosis? What is your experience with spending time in nature during cancer treatments? If applicable: What is your experience with spending time in nature after your cancer treatments?
	What outdoor experience would you be/have been comfortable with during your cancer experience? Just answer yes or no a. solitary time b. small groups c. large groups d. audio guide e. in-person guide f. zoom guide
Miscellaneous	Is there anything else you would like to share about your experience?

### Data analysis

Demographic data were analyzed using descriptive statistics, reported as means and standard deviations for continuous variables and frequencies and percentages for categorical data.

The interview transcripts were analyzed using a general inductive approach, as described by Thomas [20]. Procedures allow research findings to emerge from the frequent, dominant, or significant themes inherent in the raw data, without the restraints of previously established methodologies. The purpose of this approach was to allow for a condensed, summary format of the raw data and to establish clear links between the research questions and the summary findings derived from the data. To ensure the integrity of the findings, the transcripts were first cleaned and formatted and sent back to the participants for review. They were then read independently by two reviewers, the lead researcher (ERTO) and a research assistant trained by the investigator in qualitative analysis (MD), to gain a comprehensive understanding of the content. Following the steps outlined by Thomas [20], the analysis involved a close reading of the text to understand the details and nuances of the participants’ experiences. This was followed by creation of categories (themes) that came directly from the text to capture concepts as they appeared. Next was overlapping coding and refinement which involved repeatedly re-reading, discussing, and re-coding the data to ensure that the categories were distinct. Discrepancies were resolved through consensus-building discussions between the analyzers to enhance the credibility and trustworthiness of the analysis. Finally, continuing revision was used until no new themes emerged to ensure the final thematic groupings evolved naturally from the interviews while remaining aligned with the study's aims. The researchers imported the transcripts into NVivo software (version 1.7) to facilitate the coding process, organize the data, and provide an audit trail of the analytical decisions made throughout the study [21].

To ensure the rigor and quality of the findings, the study followed the four criteria for trustworthiness established by Lincoln and Guba [22]: credibility, transferability, dependability, and confirmability. Credibility was established through investigator triangulation, with two independent reviewers, ERTO and MD, analyzing the transcripts separately to ensure findings accurately represented the participants’ experiences; any discrepancies were resolved through iterative consensus-building discussions. To support transferability, the researchers provided “thick descriptions” of the research setting, participant demographics, and data collection processes, allowing readers to assess the applicability of the findings to other contexts. Dependability was maintained through a documented research process and the use of NVivo 1.7 software, which provided an audit trail of codes and the progression from raw data to final themes. Finally, confirmability was achieved by employing Thomas’s [20] general inductive approach to minimize researcher bias.

## Results

### Demographic data

The 15 participants were majority female (93%), diagnosed with breast cancer (60%), white (80%), and married or living with their significant other (73%). The mean age at diagnosis was 30.5 years, with a range of 22–37 years at diagnosis. The mean age at the time of consent was 32.8 years. Additional data describing the sample are depicted in Table 2.

**Table 2:** Participant demographics and clinical information

*Variable*	*Value*	*n*	*%*	*Mean*	*(SD)*		*Range*
Gender	Female	14	93				
	Male	1	7				
Education	Bachelor’s Degree or Some College	9	60				
	Advanced Degree	6	40				
Marital status	Never married	4	27				
	Living with a partner/significant other	4	27				
	Married	7	47				
Race	Black or African American	1	7				
	Asian	1	7				
	White	12	80				
	Other	1	7				
Type of cancer	Breast	9	60				
	Leukemia/Lymphoma	1	7				
	Uterine	1	7				
	Thyroid	1	7				
	Rectal	1	7				
	Rhabdomyosarcoma	1	7				
Age at diagnosis				30.5	4.6	22–27	
	18–24	3	20				
	25–29	2	13				
	30–34	7	47				
	35–39	3	20				
Age at consent				32.8	4.4	24–39	
	18–24	1	7				
	25–29	2	13				
	30–34	5	33				
	35–39	7	47				

### Overall themes

**Fig. 1:**
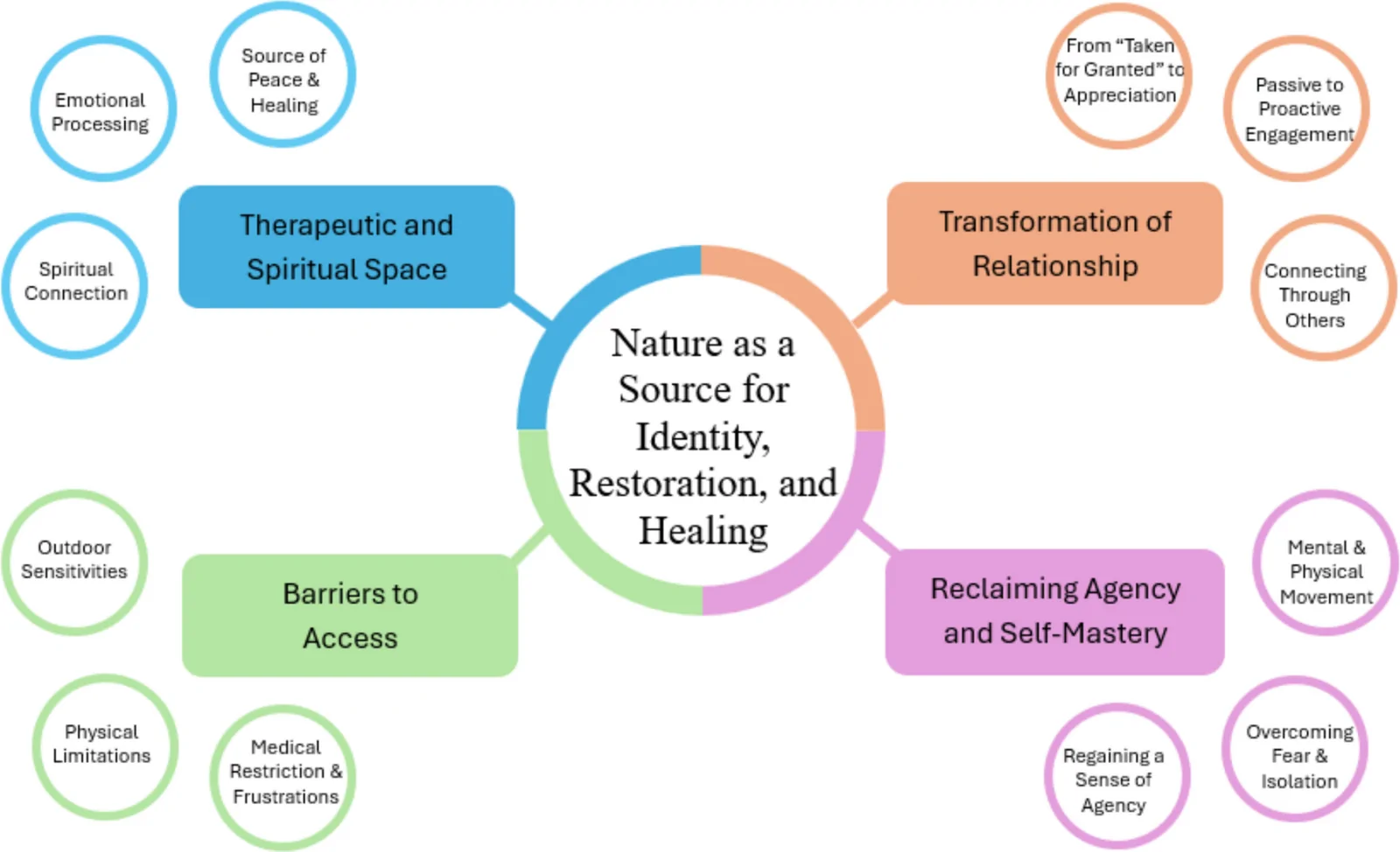
Overarching central theme, main themes and sub-themes

The qualitative analysis of the interview data revealed a complex, evolving relationship between young adult cancer survivors and the natural environment. The findings revealed an overarching central theme of Nature as a Source for Identity, Reconstruction, and Healing that is supported by four main themes and twelve sub-themes and is illustrated in Fig. 1.

First, Nature as a Therapeutic and Spiritual Space captures the role of the outdoors in emotional processing, a source of peace and healing and spiritual connection. Second, Transformation of Relationship details the shift from passive to proactive engagement with nature, from “taken for granted” to appreciations and connecting through others. Third, the role of nature in Reclaiming Agency and Self-Mastery illustrates how survivors regain a sense of agency, overcome fear and isolation and use mental and physical movement. Finally, Conflict and Barriers to Access nature addresses the medical restrictions and frustrations, physical limitations and outdoor sensitivities. (Fig. 1) and include participants’ preferences for how to engage in nature (Table 3).

**Table 3:** Meaning of nature

Nature as a Therapeutic and Spiritual Space	"I am a Christian and before diagnosis that was where I would feel closest to God. I was just like on a nature hike or I have a specific peak up the shore that I'd always go to if I was feeling stressed out about something or overwhelmed. And so that was my time to pray and to really connect to God and so I had a very positive interaction with nature." (YA11) "...so that was like that was incredible, like all of the like just the natural like the things that happened in nature, naturally, are like incredible... So just kind of like the awe and amazement level of like all of this can happen..." (YA15) “So, I really adopted I would go on a daily walk... It was a Zen experience for me, and it really felt like I was doing something in my day... It helps with my mental health hugely to have that sort of weird..." (YA03) "...the thing we very much love the most to do and we're so excited about spending the fall on the North shore in these gorgeous campgrounds and just a good thing for our souls, in general." (YA01)	
Transformation of the Relationship with Nature	"I did have a good relationship and in nature, but it was not something that I felt fully hold to the extent that I am now." (YA14) "I don't think I learned to appreciate it the same way, I do now... I know it's more of like I very much find joy from like the simple yet beautiful like naturally made things..." (YA09) "And then I don't know I just I could feel a difference after those walks in a way that just slowly seeped in." (YA08) "About the same or maybe a little bit more. I just find throughout this realizing what's important and realizing how much I enjoy that and wanting to spend more time outdoors." (YA06) "I really looked at nature, far more appreciative than I ever had before.” (YA03)	
The Role of Nature in Reclaiming Agency	"It was my saving grace... I felt alive, I felt a sense of wanting to stay alive to experience, like it was like a bigger reason to want to stay alive, to be able to experience nature." (YA13) "Once I got the diagnosis and he said 15 minutes of exercise is correlated with better outcomes, I said OKAY. I'm going to do my walk I'm, going to do you know 2 3 minutes at a time..." (YA07) "I was basically, I have to, I have to do something. So, one thing was how am I going to pass these three years. . . I'm like I have to do something to take care of myself and try and start healing myself..." (YA02) "I did, though, try to get out for a walk almost every day during treatment, just to make sure I got outside and move my body." (YA11)	
Conflict and Barriers to Nature During Illness	"I was outside for an hour... I was beet red, it was ridiculous I've never peeled like-It burned everything from an hour outside, so I tried to be a little cautious because again very white." (YA12) "I don't think that I'm comfortable enough to go out by myself, yet." (YA10) "And I went and I got really depressed because of something that happened with my disease, for the first time. I just didn't do anything and then it was finished winter where it's all cold and bone chilling." (YA04) "I mean I loved being outside nature, but I think I was much more quickly to be like ah mosquitoes or like annoyed by little things where now it's more of like I very much find joy..." (YA09) "During was sometimes harder, just depending on how I was doing. The biking dropped off for sure, I didn't travel and do as much outdoor things at that point." (YA06)	
Preferences for Future Nature Based Engagement	Small Group	“Yes, as long as there was no small talk” (YA08) “I have a lot of anxiety obstacles to deal with right now, but anything nature related I honestly think can only bring good” (YA09) “Absolutely, it's just nice to be with people who don't have to ask why you're bald, they get it” (YA15)
	Zoom Guided	“Personally no, but others could join via zoom” (YA05) “Maybe try it out but not something I’d be crazy about” (YA04) “I think the key would be if I'm feeling connected with the group” (YA14)
	Virtual Reality	“Definitely when in the hospital for long periods of time” (YA05) “Yes, but would have been difficult due to surgery” (YA06) “Yeah, you know if it's something like oh we're going on a hike through the superior national forest again that sounds really fun, it probably could have helped with my pain relief” (YA14)

#### Nature as a therapeutic and spiritual space

Nature was able to provide survivors a place to find meaning, grounding, and a feeling of being alive. For example, one participant explained: “It [nature] makes me feel like I’m doing something meaningful, honestly, like just this is life. I’m putting my hands in the dirt and I’m not sitting and staring at TV all day. I’m moving my body, it just it just feels real to me.” (YA02) Another said, “It [nature] was my saving grace. It was place where I could just go anywhere… And it was my place to go and I felt I felt good I felt okay, I felt alive, I felt a sense of wanting to stay alive to experience, like it was like a bigger reason to want to stay alive, to be able to experience nature.” (YA13).

Survivors described their time in nature as a source of mental and spiritual well-being. The data showed that being in nature provided a therapeutic routine that supports their healing. A participant said, “I just can’t believe the stillness and clarity and peace that comes from when I go on walks, even by myself, I don’t usually listen to music or I’m not playing on my phone I’m just like walking in stillness and I cannot believe the difference, it feels in my body.” (YA08) Another said, “The beauty of that place will forever be a part of me. I needed it so badly at that time in my life.” (YA03) Nature was a place where survivors could connect with God and work through the difficult emotions of cancer and feel a sense of support being outdoors. A third participant said, “I think being in nature is like one of the few places where I can really stop and do business with God and really think about like the anger and the sadness and the grief that I feel for having gone through cancer.”

#### Transformation of the relationship with nature

Participants stated that their diagnosis catalyzed a deeper connection with nature. One participant shared, “I always liked being in nature… I always enjoyed it, but I didn’t have the connection that I had after the diagnosis*.*” (YA 13) They found their cancer diagnosis caused a shift in life priorities that led to a great and desired engagement with nature. Another stated, “I just find throughout this realizing what’s important and realizing how much I enjoy that [nature] and wanting to spend more time outdoors.” (YA06) Participants transformed their relationship with nature from a simple appreciation to a deep, active interaction of nature in their daily environment. One participant stated, “Another way that I have dove into nature since being diagnosed is I have become a little bit obsessed with plants. So, like wanting to be surrounded by plants and have nature around me all the time and just so I can just sit in it and look and appreciate it.” (YA02) 

#### The role of nature in reclaiming agency

Nature was described as an active experience for the participant to take control of their emotional processing, rather than to remain a passive victim of trauma or fear. “I have been really called to spend time outdoors… I have managed to really suppress a lot of it for a long time, and I know that it’s just coming out sideways, and so I have to deal with it now and I don’t want to be fearful of cancer, for the rest of my life, I want to enjoy my life… (YA14)” Another survivor made intentional choices to assert personal agency, even when against medical teams’ directives to support their mental health and shared, “And so I was outside gardening because my hematologist said that I could last week… but it just feels like I need to hide a part of something that’s making my mental health better (YA11).” Nature was also used as a deliberate tool for self-mastery by confronting deep personal fears of being alone by choosing challenging natural environments. They shared, “I’m doing this by myself, I don’t want people to come with me. I want to go out by myself… I’m afraid of myself. I’m afraid of being alone so I’m going to go out into nature by myself. (YA02)”.

#### Barriers to nature during illness

Participants reported that time was a significant barrier during treatment where the focus was entirely on treatment, work, and basic survival which meant making time for nature non-essential. A participant stated, “What was my experience during treatments? Largely absent, I was in the gym and working and just trying to survive, I would say.” (YA14) Treatments also caused significant physical barriers, fatigue, chemotherapy effects, that required a significant reduction in activity. Another participant shared, “So we just didn’t do much, we had to kind of scale it back, we stayed in the cabin most of it. We did not hike, we walked a little bit, but it’s just not what we would have done, had I been healthy.” (YA01) Sensitivity to the outdoor element like sun and cold exposure transformed a previously enjoyable experience into a source of stress and fear. Another participant acknowledged, “I’m a very fair skinned individual and now have to be extra cautious when it comes to being the sun…. Now if I get a sunburn, I’m like, oh my gosh, I got a sunburn. My doctors are all mad, that’s sort of a negative thing.” (YA03) 

#### Preferences for nature engagement

Participants preferred to experience nature engagement with others, and many expressed interests in a group setting. Participants reported being interested in a small group of 4–5 people, and in a large group of 8–10 people while in nature, but also enjoyed solitary experiences. The least preferred modality of engagement was reported to be Zoom guided, one participant stated “I think the key would be if I’m feeling connected with the group” which further indicates the desire for connection within a group. Overall, participants preferred in-person nature engagement experiences and wanted to have experiences that supported connection to a group.

## Discussion

This study is the first to describe the lived experience of YA cancer survivors with nature. Previous studies have focused on specific nature-based interventions rather than the lived experience of YA cancer survivors within nature. These results revealed an overall central theme of Nature as a Source for Identity, Reconstruction, and Healing and four main themes of that experience: Nature as a therapeutic and spiritual space, a transformation of their relationship with nature post-diagnosis, its role in reclaiming agency over their lives, and the conflicts and barriers encountered during survivorship. Results suggest that nature is where survivors are able to reconcile their pre- and post-diagnosis identities.

Consistent with previous studies, participants in this study viewed nature as a therapeutic and spiritual space that provides opportunity for mental clarity, reflection, self-care, and healing during a physical and mentally challenging time [12]. Previous studies support nature as a sacred and available resource for coping with cancer, regardless of religion, philosophy, age, or gender [23] and reported nature as the most important resource for cancer survivors in coping with their cancer [13]. Studies of primarily female cancer survivors show that women in their late 20 s report higher levels of nature connectedness than males of the same age [24].

In this study, survivors explained how their relationship with nature was transformed, deepened, and intensified after their diagnosis. Their cancer diagnosis was a catalyst to a profound and deeper connection with nature, with a clear before-and-after delineation. Additional information from this study that adds to the current literature was that nature had a significant impact on reclaiming agency. By choosing to interact with nature, participants took control of their emotional processing rather than giving in to fear and anger and took control over their body and routine. One participant actually “prescribed” being in nature as a therapy to herself as she recognized its healing effects.

Other new findings identified barriers to being in nature, such as cold weather, sun exposure, and fear of tick-borne diseases, which created feelings of being unsafe, anxious, and even panic, which expands previous research reports regarding basic safety needs within nature [25]. These perceived barriers added to psychological and physical limitations felt in other areas of the participants’ lives that spilled over into the outdoors, where they previously felt safe and relaxed. To mitigate these challenges, nature-based virtual reality could offer a controlled alternative, providing the restorative benefits of natural environments without the physical risks or psychological stressors.

The results were consistent with those of studies of healthy people who also preferred being in small group nature engagement sessions. Outdoor walking, especially with a group, can lead to increased psychosocial health reported as an increase in happiness, nature relatedness, and a higher quality of life [26, 27], which is also relevant to YA cancer survivors who may want to connect with others who have shared cancer experiences for emotional and informational support [28]. There was some hesitation for large group engagement activity due to the COVID-19 pandemic. Pandemic-related burnout also influenced participants’ response to their preference for a video web conference nature engagement. “Zoom fatigue” is a term widely used to describe the inability to focus and decreased responsiveness when it comes to web conference-based style activities, and this was an explanation given by some participants in their choice of preferences [29].

The results of this study could be used to inform the development of a nature-based intervention for YA cancer survivors. One of the most common complications of cancer for young adults is loneliness and isolation [30]. Along with findings of this study that YA cancer survivors look to nature for promoting well-being, there are emerging frameworks to guide the design of future studies aiming to investigate the relationship between socially restorative needs of YA cancer survivors and ecosystems. One example is the Ecosocial Partnership Framework, which describes an approach aimed at fostering a sense of ecosocial restoration, adaptability, and resilience [31].

### Limitations

Qualitative research has small sample sizes and the findings may not be generalizable to the broader population. Data were collected from interviews where participants may provide responses they perceive as acceptable to the researchers. While steps were taken to reduce bias, the background and experiences of the researchers may have influenced the interpretation of the data.

## Conclusions

YA cancer survivors described nature as a source of clarity, healing, and connection, with their diagnosis often prompting a deeper, more active engagement with nature. However, physical limitations, fatigue, fear of adverse effects, and sensitivity to the outdoors presented barriers for outdoor nature engagement. Participants stated a preference for in-person, small-group nature engagement for social connection and shared experience with others who understand their journey. Overall, the findings support nature’s vital role as a resource for emotional and physical recovery and encourage more research into modalities of nature-based interventions that facilitate social interaction, reduce barriers to nature engagement, and promote well-being for this population.

## Data Availability

No datasets were generated or analysed during the current study.

## Declarations

### Public involvement statement

No public involvement in any aspect of this research.

### Guidelines and standards statement

This manuscript was drafted against the Consolidated criteria for reporting qualitative studies (COREQ) [32] for qualitative research.

### Use of artificial intelligence

AI or AI-assisted tools were not used in drafting any aspect of this manuscript.
